# Financial constraint and perceptions of COVID-19

**DOI:** 10.1038/s41598-023-30118-9

**Published:** 2023-03-01

**Authors:** Jennifer S. Trueblood, Abigail B. Sussman, Daniel O’Leary, William R. Holmes

**Affiliations:** 1 Department of Psychological and Brain Sciences and Cognitive Science Program, Indiana University, Bloomington, IN USA; 2 Booth School of Business, University of Chicago, Chicago, IL USA; 3 Cognitive Science Program and Department of Mathematics, Indiana University, Bloomington, IN USA

**Keywords:** Human behaviour, Risk factors

## Abstract

In early March 2020, two crises emerged: the COVID-19 public health crisis and a corresponding economic crisis resulting from business closures and skyrocketing job losses. While the link between socioeconomic status and infectious disease is well-documented, the psychological relationships among economic considerations, such as financial constraint and economic anxiety, and health considerations, such as perceptions of disease spread and preventative actions, is not well understood. Despite past research illustrating the strong link between financial fragility and a wide range of behaviors, surprisingly little research has examined the psychological relationship between the economic crisis and beliefs and behaviors related to the co-occurring health crisis. We show that financial constraint predicts people’s beliefs about both their personal risk of infection and the national spread of the virus as well as their social distancing behavior. In addition, we compare the predictive utility of financial constraint to two other commonly studied factors: political partisanship and local disease severity. We also show that negative affect partially mediates the relationship between financial constraint and COVID-19 beliefs and social distancing behaviors. These results suggest the economic crisis created by COVID-19 spilled over into people’s beliefs about the health crisis and their behaviors.

## Introduction

Following the widespread identification of COVID-19 in the US in March of 2020, experts estimated that there could be millions of cases of COVID-19 in the US in the coming years. In reaction to the rapid disease spread, national and state stay-at-home orders led many businesses to close and lay off employees in 2020. These business closures led to a separate economic crisis, with the unemployment rate reaching 14.7% in April 2020, higher than at any other point since the Great Depression. Rather than existing in parallel, these two crises are inextricably linked in many ways. While the association between socioeconomic status (SES) and infectious disease is well-documented^1–3^, the psychological relationships among economic considerations, such as financial constraint and economic anxiety, and health considerations, such as perceptions of disease spread and preventative behaviors, is not well understood.

Beliefs about the severity of the spread of COVID-19 and one’s own likelihood of being infected have implications for individual behavior and consequently for the trajectory of the virus. Indeed, epidemiological models of disease control use such beliefs as predictors^4^, and these beliefs are thought to motivate preventative behaviors^5,6^. However, the factors that contribute to these beliefs are not fully understood. Many epidemiological models assume that people’s risk perceptions and preventative behaviors are determined by disease prevalence or some proxy for it^7–11^. In contrast, the media highlights the role of political affiliation as a central factor, a finding supported by recent academic literature^12–14^. We propose that financial constraint (defined as an individual’s subjective feelings about the limits of their personal finances) and economic anxiety associated with COVID-related job losses and pay cuts has been an overlooked, yet important predictor of people’s risk perceptions, beliefs about disease spread, and preventative behaviors during the early days of the pandemic in 2020.

Individuals with lower income or SES may be at greater risk of contracting illnesses such as COVID-19^1–3^. However, we propose that the psychological relevance of financial constraint and economic anxiety extends beyond increased susceptibility of contracting the disease. Financial fragility has been shown to impact cognition and behavior in a range of ways^15–17^. For example, financial scarcity has been shown to lead to increased impulsivity and risk-taking^18^, to contribute to overborrowing^17^, and to shifts in moral standards^19^. The effects of financial scarcity are also highly consequential in organizational contexts^20–22^. Similar to other stressors outside of the workplace, such as metal health and family roles^23,24^, financial scarcity is known to hinder workplace performance^22^. Based on this past research illustrating the strong link between financial fragility and a wide range of behaviors, surprisingly little psychological research has examined this issue in the context of the COVID-19 pandemic.

The goal of this paper is to illustrate the relationship between financial constraint, associated with job losses and pay cuts due to COVID-19, and perceptions of COVID-19 during the early days of the pandemic. Specifically, we show that financial constraint corresponds not only to higher perceptions of one’s own risk of contracting the virus, but also to higher perceptions of the spread of the virus across the US and to increased social distancing behavior in Spring 2020. Why might financial constraint correspond to increased estimates of disease spread and social distancing? One potential pathway is through emotional distress. Individuals with lower levels of SES are more likely than individuals with high SES to experience emotional distress^25,26^. This is true for multiple measures of both SES, including income and education^25^, as well as occupation^27^, and emotional distress, including self-report and biological assessments^25,26,28^. In particular, a person’s financial well-being has been shown to play a causal role in the relationship between SES and emotional distress^29^. Although there are multiple pathways through which a person’s financial well-being might affect his or her emotional state, recent work suggests that concern with one’s current and future financial situation directly impacts affective well-being^30^.

A person’s emotional state plays a key role in shaping his or her judgments and decisions^31,32^. Importantly, emotion uniquely impacts assessments of risk^33–37^. In a seminal paper^36^, Johnson and Tversky demonstrated that exposure to an incidental negative emotion induction led to higher estimates of the incidence of many types of negative events (e.g., natural disasters, terrorist attacks), even though these events were unrelated to the content of the induction. More recent studies have shown that it is not only the valence of the emotional state (negative versus positive) that matters^38,39^. Rather, emotional states accompanied by appraisals of uncertainty and lack of control are more likely to lead to perception of increased risk than emotional states that lack these features^31,38,39^.

This research illustrates a strong psychological relationship between people’s experiences due to the economic crisis and their perceptions of the co-occurring COVID-19 health crisis in Spring 2020. In this paper, we first sought to quantify the relationship between COVID-related job impacts and financial constraint. Next, we examined the relationships between financial constraint, predictions about one’s own risk of contracting the virus, beliefs about the national spread of the virus in the US, and social distancing behavior. Finally, we explored whether negative affect (defined as a person’s subjective experience of negative emotions) mediated the relationship between financial constraint and beliefs about COVID-19 as well as social distancing behavior. To understand these questions, we analyzed two cross-sectional waves of a nationally representative survey, reaching over 2,500 respondents overall (and replicated in two additional waves with over 2,300 respondents in the supplement).

## Results

### Predictors of financial constraint

Our key objective is testing the hypothesis that increased financial constraint is related to heightened predictions about the spread of COVID-19 and increased social distancing behavior in Spring 2020. To begin, it is important to establish the factors associated with financial constraint and the extent to which impacts of COVID-19 on employment correspond to overall financial constraint. It seems reasonable to believe that perceptions of financial constraint are directly related to income, where lower income leads to increased feelings of financial constraint. However, our goal is to determine whether financial constraint was closely related to COVID-19 job impacts at the start of the pandemic. Specifically, we examine two ways that COVID-19 has influenced people’s jobs. First, the COVID-19 pandemic has made it more difficult for people to earn money. Second, many people’s jobs have increased their risk of contracting COVID-19. In addition to these two factors, we also include the following control factors: income, age, education, and political affiliation.

**Figure 1:**
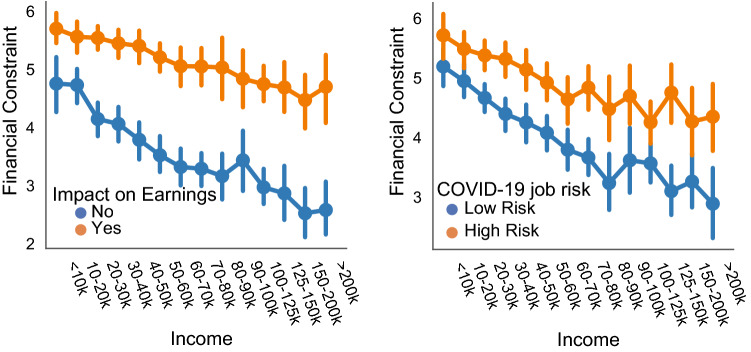
Relationship between financial constraint, income, and COVID-19 job impacts. Financial constraint was measured on a 7 point scale. In the left panel, the impact on earnings variable was measured on a 5 point scale, but recoded in the figure for illustrative purposes. Similarly, in the right panel, the COVID-19 job risks variable was measured on a 5 point scale, but recoded for plotting purposes. Error bars show the 95% confidence interval.

An ordinal logistic regression was calculated to predict financial constraint based on the six predictors mentioned above: impact on earnings, COVID-19 job risks, income, age, education, and political affiliation. For all regression models, political affiliation was treated as nominal. All analyses were performed in Jamovi. A significant regression equation was found ($\chi ^2(9,2682)$ = 856, p < .001), with $R^2_{McF} = 0.084$. As shown in the Table 1, impact on earnings, COVID-19 job risks, and income are all significant predictors of financial constraint. Specifically, people who report negative impacts of COVID-19 on their ability to earn money also report greater feelings of financial constraint. Increased job risks associated with COVID-19 are also related to increased feelings of financial constraint. As shown in Fig. 1, the relationship between financial constraint and the two COVID-19 job variables persists across the entire income spectrum.

**Table 1:** Model coefficients for ordinal logistic regression predicting feelings of financial constraint.

Predictor	Estimate	95% CI Lower	95% CI Upper	SE	Z	p	Odds ratio
Impact on Earnings	0.82459	0.74071	0.9092	0.04298	19.188	<.001	2.281
Job Risks	0.19236	0.14094	0.2439	0.02627	7.324	<.001	1.212
Income	−0.13878	−0.15908	−0.1186	0.01033	−13.429	<.001	0.87
Age	−0.00406	−0.00827	1.53E−04	0.00215	−1.889	0.059	0.996
Education	0.0113	−0.02741	0.05	0.01975	0.572	0.567	1.011
Political Affiliation							
Lean Rep—Rep	−0.052	−0.31277	0.2089	0.13304	−0.391	0.696	0.949
Ind—Rep	0.07501	−0.11848	0.2686	0.09872	0.76	0.447	1.078
Lean Dem—Rep	−0.07461	−0.29415	0.1449	0.112	−0.666	0.505	0.928
Dem—Rep	0.1796	−0.00942	0.3687	0.09646	1.862	0.063	1.197

We also compared the standard ordinal logistic regression model (i.e., proportional odds model) with the partial proportional odds model and heteroskedastic ordered logisitic regression model because the Brant Test^40^ and the Breusch-Pagan test showed violations of the parallel lines assumption and homoscedasticity respectively^41^. Similar to the proportional odds model, these alternative models showed that impact on earnings, COVID-19 job risks, and income are all significant predictors of financial constraint. Model comparisons showed that the AIC values were higher (worse) for the proportional odds model (AIC = 9377) as compared to the partial proportional odds model (AIC = 9354). However, the BIC values were lower (better) for the proportional odds model (BIC = 9465) as compared to the partial proportional odds model (BIC = 9501). The AIC values were slightly lower (better) for the proportional odds model (AIC = 9377) as compared to the heteroskedastic model (AIC = 9378). Additionally, the BIC values were lower (better) for the proportional odds model (BIC = 9465) as compared to the heteroskedastic model (BIC = 9502). Thus, one model is not clearly superior to the others and the key conclusions do not depend on the selection of model. Stata code^42^ to produce these results are on OSF.

To assess whether the impact on earnings and COVID-19 job risk variables significantly improve the fit of the model, we fit two additional ordinal logistic regression models (i.e., proportional odds models) where these variables are omitted. A significant regression equation was found when omitting impact on earnings ($\chi ^2(8,2682) = 467$, $\hbox {p }< .001$), with $R^2_{McF} = 0.046$. Likewise, a significant regression equation was found when omitting COVID-19 job risks ($\chi ^2(8,2682)$ = 802, p < .001), with $R^2_{McF} = 0.079$. However, both the AIC and BIC values were higher (worse) for the two reduced models (omitting impact on earnings: AIC = 9763, BIC = 9846; omitting job risks: AIC = 9429, BIC = 9511) as compared to the full model (AIC = 9377, BIC = 9465), suggesting the full model provides the best account of the data.

### Predictors of beliefs about personal risk of contracting COVID-19

In this section, we examine the factors associated with people’s beliefs about their own personal risk of being infected by COVID-19 at the start of the pandemic. To begin, beliefs about personal risk are highly correlated with beliefs about the national spread of the virus (r = 0.601, $\hbox {p }< .001$). However, participants, in Spring 2020, believed the percentage of people in the US that will be infected within the next year (M = 37.1%) was greater than the likelihood that they will be infected within the next year (M = 27.5%; t(2681) = -20.681, $\hbox {p }< .001$). This suggests that people were more optimistic about their own prospects of contracting COVID-19 as compared to others.

While there are many factors that are likely related to people’s beliefs about their personal risk and the national spread of the disease as well as their social distancing behavior (the latter two variables are described in subsequent sections), we focus on three key factors: financial constraint, political affiliation, and local disease severity. Political affiliation and local disease severity have received significant attention in both the media and in academic work. In particular, the behavioral epidemiology literature suggests that people’s beliefs and behaviors are linked to local disease prevalence^7–11^. To account for effects of the local severity of COVID-19 where each participant resides, we calculated the local cases per capita associated with each person at the time of data collection. To do this, we calculated the number of COVID-19 cases near them (using the NYTimes county level data https://github.com/nytimes/covid-19-data) divided by the population size (from 2010 US Census data). Specifically, for the zip code associated with each participant, we found all counties that have any geographic portion that lies within 50 miles of that zip code. We then summed the reported COVID-19 cases and total population in those counties to calculate the local case density. In the regression models described below, we used the log of the local case density because these numbers tended to be very small and model estimation was improved by transforming these values by the log.

In addition to the factors mentioned above, we also include the following control factors: COVID-19 job risks, income, age, and education. The COVID-19 job risks question is a critical control because relationships between financial constraint and COVID-19 beliefs and social distancing behaviors might simply be driven by people’s job situation. That is, if a person’s job put them at risk of contracting COVID-19 at work, it seems reasonable to believe that they would report increased personal risk and less social distancing.

For beliefs about personal risk, an ordinal logistic regression was calculated with the seven predictors described above: financial constraint, political affiliation, local cases, COVID-19 job risks, income, age, and education. We opted to use ordinal logistic regression because responses to the personal risk question were on a 21-point scale (as described in the Methods) and thus not continuous. Additionally, the objective distance between response options was not equal. For example, the distance between the first two response options was 0.5% whereas the distance between the third and fourth response options was 1% and the distance between the 12th and 13th response options was 10% (see supplemental materials for the full list of response options). Additionally, it is well known that people’s perception of probabilities is nonlinear^43^. Thus, the subjective distance between categories is unknown, but we can still rank the values. (Note that we observed violations of normality as determined by the Shapiro-Wilk normality test (W = 0.915, $\hbox {p }< .001$) and we found evidence of heteroskedasticity as determined by the Breusch-Pagan test (BP = 38.287, $\hbox {p }< .001$). Thus standard linear regression would be inappropriate for these reasons as well.)

**Figure 2:**
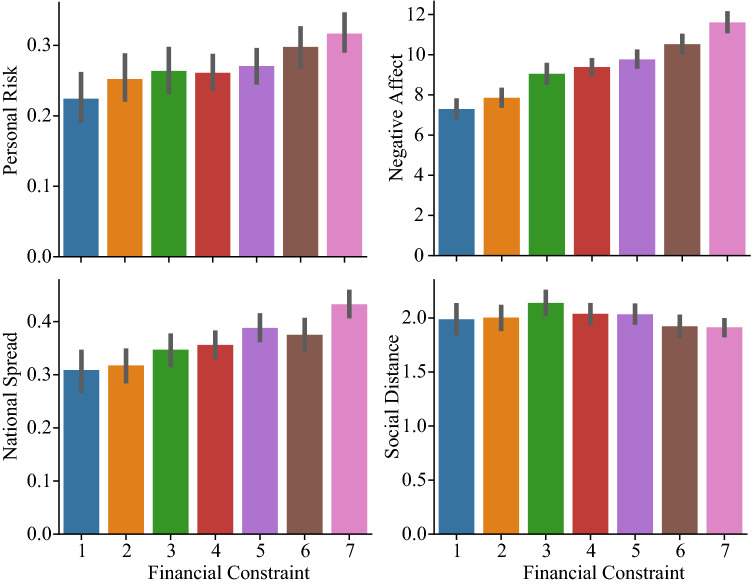
Relationship between financial constraint and key variables of interest. Top left: relationship between financial constraint and beliefs about one’s risk of contracting COVID-19. Top right: relationship between financial constraint and negative affect, measured using the short-form of the PANAS. Bottom left: relationship between financial constraint and beliefs about the national spread of COVID-19. Bottom right: relationship between financial constraint and social distancing behavior, where smaller values on the social distancing variable indicted increased social distancing behavior. Error bars show the 95% confidence interval.

A significant regression equation was found ($\chi ^2(10,2682)$ = 124, p < .001), with $R^2_{McF} = 0.009$. As shown in Table 2, financial constraint, COVID-19 job risks, income, age, and political affiliation are all significant predictors of people’s beliefs of their personal risk of contracting COVID-19. Specifically, increased financial constraint is associated with an increased belief in one’s personal risk of contracting COVID-19, as illustrated in the top left panel of Fig. 2. Self identified Democrats also have heightened perceptions of personal risk as compared to self identified Republicans. Interestingly, local disease severity is not a significant predictor.

**Table 2:** Model coefficients for ordinal logistic regression predicting beliefs about personal risk.

Predictor	Estimate	95% CI Lower	95% CI Upper	SE	Z	p	Odds ratio
Financial Constraint	0.08836	0.04963	0.1272	0.01978	4.4676	<.001	1.092
Job Risks	0.07975	0.03109	0.1283	0.02483	3.2115	0.001	1.083
Income	0.06202	0.04176	0.0823	0.01034	5.9951	<.001	1.064
Age	−0.00459	−0.00862	−0.00056	0.00206	−2.2288	0.026	0.995
Education	0.02665	−0.01129	0.0646	0.01936	1.3771	0.168	1.027
Local Cases (log)	0.00159	−0.16156	0.1647	0.08323	0.0191	0.985	1.002
Political Affiliation							
Lean Rep—Rep	0.21897	−0.03842	0.4765	0.13134	1.6672	0.095	1.245
Ind—Rep	0.20993	0.02037	0.3997	0.09675	2.1698	0.03	1.234
Lean Dem—Rep	0.47555	0.26058	0.6908	0.10974	4.3334	<.001	1.609
Dem—Rep	0.41177	0.22791	0.5959	0.09387	4.3864	<.001	1.509

We also compared the standard ordinal logistic regression model (i.e., proportional odds model) with the partial proportional odds model and heteroskedastic ordered logisitic regression model because the Brant Test^40^ and the Breusch-Pagan test showed violations of the parallel lines assumption and homoscedasticity respectively^41^. For these analyses, we combined some of the response options to reduce the number of categories from 21 to 12 to overcome convergence issues with the alternative models. Similar to the proportional odds model, these alternative models showed that financial constraint, COVID-19 job risks, income, age, and political affiliation are all significant predictors of people’s beliefs of their personal risk of contracting COVID-19. Model comparisons showed that the AIC values were higher (worse) for the proportional odds model (AIC = 11827) as compared to the partial proportional odds model (AIC = 11816). However, the BIC values were lower (better) for the proportional odds model (BIC = 11951) as compared to the partial proportional odds model (BIC = 12116). Likewise, the AIC values were higher (worse) for the proportional odds model (AIC = 11827) as compared to the heteroskedastic model (AIC = 11821). However, the BIC values were lower (better) for the proportional odds model (BIC = 11951) as compared to the heteroskedastic model (BIC = 11962). Thus, one model is not clearly superior to the others and the key conclusions do not depend on the selection of model. Stata code^42^ to produce these results are on OSF.

To assess whether the financial constraint and political affiliation variables significantly improve the fit of the model, we fit two additional ordinal logistic regression models (i.e., proportional odds models) where these variables are omitted. A significant regression equation was found when omitting financial constraint ($\chi ^2(9,2682) = 104$, $\hbox {p }< .001$), with $R^2_{McF} = 0.007$. However, the AIC and BIC values were higher (worse) for the reduced model omitting financial constraint (AIC = 14331, BIC = 14502) as compared to the full model (AIC = 14313, BIC = 14490), suggesting the full model including financial constraint provides a better accounting of the data. Likewise, a significant regression equation was found when omitting political affiliation ($\chi ^2(6,2682)$ = 96.9, p < .001), with $R^2_{McF} = 0.007$. In this case, the AIC value was higher for the reduced model omitting political affiliation (AIC = 14332, BIC = 14485) as compared to the full model (AIC = 14313, BIC = 14490). However, the BIC value was higher for the full model as compared to the reduced model, thus model selection is less clear in this case.

We hypothesized that negative affect mediates the relationship between financial constraint and beliefs about personal risk of contracting COVID-19. Negative affect was calculated as the sum of the five negative affect items on the short-form of the PANAS (i.e., upset, hostile, ashamed, nervous, and afraid). The indirect and total effects from the mediation analysis are shown in Table 3. As shown in the table, the standardized regression coefficient between financial constraint and negative affect was statistically significant ($\beta$ = .296, z = 16.05, p < .001), as illustrated in Fig. 2. The standardized regression coefficient between negative affect and personal risk ($\beta$ = .162, z = 8.12, p < .001) was also significant. The standardized indirect effect was (.296)(.162) = .048, which was significant (z = 7.25, p < .001).

**Table 3:** Indirect and total effects for personal risk mediation model.

Type	Effect	Estimate	SE	95% CI	95% CI	$\beta$	z	p
				Lower	Upper			
Indirect	Financial Const. $\Rightarrow$ Neg PANAS $\Rightarrow$ Personal Risk	0.1526	0.0211	0.1114	0.194	0.0479	7.25	<.001
Component	Financial Const. $\Rightarrow$ Neg PANAS	0.6842	0.0426	0.6006	0.768	0.296	16.05	<.001
	Neg PANAS $\Rightarrow$ Personal Risk	0.2231	0.0275	0.1693	0.277	0.1618	8.12	<.001
Direct	Financial Const. $\Rightarrow$ Personal Risk	0.0798	0.0635	−0.0447	0.204	0.025	1.26	0.209
Total	Financial Const.$\Rightarrow$ Personal Risk	0.2324	0.0614	0.1121	0.353	0.0729	3.79	<.001

Confidence intervals computed with method: Standard (Delta method).

Betas are completely standardized effect sizes.

### Predictors of beliefs about the national spread of COVID-19

Next, we examined the factors that predict people’s beliefs about the national spread of COVID-19 using ordinal regression. The same response scale was used for both the personal risk and national spread questions. Thus the same rationale for the use ordinal logistic regression applies here. (We also observed violations of normality as determined by the Shapiro-Wilk normality test (W = 0.965, p < .001) and we found evidence of heteroskedasticity as determined by the Breusch-Pagan test (BP = 25.194, p < .001). Thus, standard linear regression is not appropriate for these reasons as well.) Similar to the analyses described above, we examine seven predictors: financial constraint, political affiliation, local cases, COVID-19 job risks, income, age, and education. A significant regression equation was found ($\chi ^2(10,2682)$ = 103, p < .001), with $R^2_{McF} = 0.007$. As shown in Table 4, financial constraint, age, education, and political affiliation are all significant predictors of people’s beliefs about the spread of COVID-19. Specifically, increased financial constraint is associated with higher predicted US infection rates, as illustrated in Fig. 2. Self identified Democrats also report a higher proportion of the US will be infected as compared to self identified Republicans. As before, local disease severity is not a significant predictor.

**Table 4:** Model coefficients for ordinal logistic regression predicting beliefs about national spread.

Predictor	Estimate	95% CI Lower	95% CI Upper	SE	Z	p	Odds ratio
Financial Constraint	0.11554	0.07706	0.15409	0.01965	5.879	<.001	1.122
Job Risks	0.04359	−0.00442	0.09163	0.0245	1.779	0.075	1.045
Income	0.01874	−0.00129	0.03879	0.01023	1.833	0.067	1.019
Age	−0.0061	−0.01013	−0.00208	0.00205	−2.971	0.003	0.994
Education	−0.03935	−0.07723	−0.0015	0.01932	−2.037	0.042	0.961
Local Cases (log)	−0.07418	−0.23517	0.08683	0.08214	−0.903	0.366	0.929
Political Affiliation							
Lean Rep—Rep	0.03837	−0.22278	0.29952	0.13321	0.288	0.773	1.039
Ind—Rep	0.13958	−0.05151	0.33077	0.09751	1.431	0.152	1.15
Lean Dem—Rep	0.38131	0.16573	0.59718	0.11005	3.465	<.001	1.464
Dem—Rep	0.3877	0.20426	0.57141	0.09366	4.139	<.001	1.474

We also compared the standard ordinal logistic regression model (i.e., proportional odds model) with the partial proportional odds model and heteroskedastic ordered logisitic regression model because the Brant Test^40^ and the Breusch-Pagan test showed violations of the parallel lines assumption and homoscedasticity respectively^41^. For these analyses, we combined some of the response options to reduce the number of categories from 21 to 12 to overcome convergence issues with the alternative models. Similar to the proportional odds model, these alternative models showed that financial constraint, age, education, and political affiliation are all significant predictors of people’s beliefs about the spread of COVID-19. Model comparisons showed that the AIC values were lower (better) for the proportional odds model (AIC = 12148) as compared to the partial proportional odds model (AIC = 12151). The BIC values were also lower (better) for the proportional odds model (BIC = 12272) as compared to the partial proportional odds model (BIC = 12393). The AIC values were higher (worse) for the proportional odds model (AIC = 12148) as compared to the heteroskedastic model (AIC = 12144). However, the BIC values were lower (better) for the proportional odds model (BIC = 12272) as compared to the heteroskedastic model (BIC = 12279). Thus, one model is not clearly superior to the others and the key conclusions do not depend on the selection of model. Stata code^42^ to produce these results are on OSF.

To assess whether the financial constraint and political affiliation variables significantly improve the fit of the model, we fit two additional ordinal logistic regression models (i.e., proportional odds models) where these variables are omitted. A significant regression equation was found when omitting financial constraint ($\chi ^2(9,2682)$ = 68.2, p < .001), with $R^2_{McF} = 0.005$. However, the AIC and BIC values were higher (worse) for the reduced model omitting financial constraint (AIC = 14350, BIC = 14521) as compared to the full model (AIC = 14317, BIC = 14494), suggesting the full model with financial constraint provides a better accounting of the data. Likewise, a significant regression equation was found when omitting political affiliation ($\chi ^2(6,2682)$ = 78.7, p < .001), with $R^2_{McF} = 0.005$. In this case, the AIC value was higher for the reduced model omitting political affiliation (AIC = 14333, BIC = 14486) as compared to the full model (AIC = 14317, BIC = 14494). However, the BIC value was higher for the full model as compared to the reduced model, thus model selection is less clear in this case.

Similar to before, we hypothesized that negative affect mediates the relationship between financial constraint and beliefs about the national spread of COVID-19. Negative affect was calculated as before. The indirect and total effects from the mediation analysis are show in Table 5. As shown in the table, the standardized regression coefficient between financial constraint and negative affect was statistically significant ($\beta$ = .296, z = 16.05, p < .001), as was the standardized regression coefficient between negative affect and national spread ($\beta$ = .102, z = 5.12, p < .001). The standardized indirect effect was (.296)(.102) = .030, which was significant (z = 4.87, p < .001).

**Table 5:** Indirect and total effects for national spread mediation model.

Type	Effect	Estimate	SE	95% CI	95% CI	$\beta$	z	p
				Lower	Upper			
Indirect	Financial Const. $\Rightarrow$ Neg PANAS $\Rightarrow$ Nat. Spread	0.0727	0.0149	0.0434	0.102	0.0302	4.87	<.001
Component	Financial Const. $\Rightarrow$ Neg PANAS	0.6842	0.0426	0.6006	0.768	0.296	16.05	<.001
	Neg PANAS $\Rightarrow$ Nat. Spread	0.1062	0.0208	0.0655	0.147	0.1021	5.12	<.001
Direct	Financial Const. $\Rightarrow$ Nat. Spread	0.2324	0.048	0.1383	0.327	0.0966	4.84	<.001
Total	Financial Const. $\Rightarrow$ Nat. Spread	0.3051	0.0461	0.2148	0.395	0.1268	6.62	<.001

Confidence intervals computed with method: Standard (Delta method).

Betas are completely standardized effect sizes.

### Predictors of social distancing

In our final set of analyses, we examined the factors that predict people’s social distancing behavior using ordinal logistic regression. Similar to before, we examine seven predictors: financial constraint, political affiliation, local cases, COVID-19 job risks, income, age, and education. A significant regression equation was found ($\chi ^2(10,2682)$ = 70.3, p < .001), with $R^2_{McF} = 0.011$. As shown in Table 6, financial constraint, COVID-19 job risks, the percentage of local cases, and political affiliation, are all significant predictors of people’s social distancing behavior. Specifically, increased financial constraint is associated with increased social distancing, as illustrated in Fig. 2. Self identified Democrats also report more social distancing as compared to self identified Republicans. Unlike the analyses examining beliefs about personal risk and national spread of COVID-19, a higher proportion of local cases is associated with greater social distancing.

**Table 6:** Model coefficients for ordinal logistic regression predicting social distancing.

Predictor	Estimate	95% CI Lower	95% CI Upper	SE	Z	p	Odds ratio
Financial Constraint	−0.07902	−0.12032	−0.03777	0.02106	−3.753	<.001	0.924
Job Risks	0.14096	0.08839	0.19366	0.02685	5.25	<.001	1.151
Income	−0.02151	−0.04322	1.66E−04	0.01107	−1.944	0.052	0.979
Age	−0.00052	−0.00488	0.00384	0.00222	−0.234	0.815	0.999
Education	0.00425	−0.03651	0.04505	0.02081	0.204	0.838	1.004
Local Cases (log)	−0.26096	−0.43581	−0.08645	0.08911	−2.929	0.003	0.77
Political Affiliation							
Lean Rep—Rep	0.10844	−0.16636	0.38286	0.14009	0.774	0.439	1.115
Ind—Rep	−0.31647	−0.52049	−0.11276	0.104	−3.043	0.002	0.729
Lean Dem—Rep	−0.3743	−0.60624	−0.1429	0.11818	−3.167	0.002	0.688
Dem—Rep	−0.32864	−0.52536	−0.13224	0.10027	−3.277	0.001	0.72

We also compared the standard ordinal logistic regression model (i.e., proportional odds model) with the partial proportional odds model and heteroskedastic ordered logisitic regression model because the Brant Test^40^ and the Breusch-Pagan test showed violations of the parallel lines assumption and homoscedasticity respectively^41^. Similar to the proportional odds model, these alternative models showed that financial constraint, COVID-19 job risks, the percentage of local cases, and political affiliation, are all significant predictors of people’s social distancing behavior. Model comparisons showed that the AIC values were higher (worse) for the proportional odds model (AIC = 6544) as compared to the partial proportional odds model (AIC = 6533). However, the BIC values were lower (better) for the proportional odds model (BIC = 6627) as compared to the partial proportional odds model (BIC = 6668). The AIC values were higher (worse) for the proportional odds model (AIC = 6544) as compared to the heteroskedastic model (AIC = 6540). However, the BIC values were lower (better) for the proportional odds model (BIC = 6627) as compared to the heteroskedastic model (BIC = 6652). Thus, one model is not clearly superior to the others and the key conclusions do not depend on the selection of model. Stata code^42^ to produce these results are on OSF.

To assess whether the financial constraint, political affiliation, and local cases variables significantly improve the fit of the model, we fit three additional ordinal logistic regression models (i.e., proportional odds models) where these variables are omitted. A significant regression equation was found when omitting financial constraint ($\chi ^2(9,2682) = 56.2$, $\hbox {p }< .001$), with $R^2_{McF} = 0.009$. Likewise, a significant regression equation was found when omitting political affiliation ($\chi ^2(6,2682) = 47.1$, $\hbox {p }< .001$), with $R^2_{McF} = 0.007$. Finally, a significant regression equation was found when omitting the local cases variable ($\chi ^2(9,2682) = 61.5$, $\hbox {p }< .001$), with $R^2_{McF} = 0.009$. Examining the AIC and BIC values (reduced model omitting financial constraint: AIC = 6552, BIC = 6629; reduced model omitting political affiliation: AIC = 6555, BIC = 6614; reduced model omitting local cases: AIC = 6549, BIC = 6626; full model: AIC = 6540, BIC = 6623) revealed that the full model was preferred to the model omitting financial constraint and the model omitting the local cases (although the differences in AIC and BIC values were small). The model selection is less clear when comparing the full model to the model omitting political affiliation.

Similar to beliefs about personal risk and the national spread of COVID-19, we hypothesized that negative affect mediates the relationship between financial constraint and social distancing behavior. Negative affect was calculated as before. The indirect and total effects from the mediation analysis are show in Table 7. As shown in the table, the standardized regression coefficient between financial constraint and negative affect was statistically significant ($\beta = .296$, $\hbox {z }= 16.05$, $\hbox {p }< .001$), as was the standardized regression coefficient between negative affect and social distancing ($\beta = -0.056$, $\hbox {z }= -2.77$, $\hbox {p }= .006$). The standardized indirect effect was (.296)(-0.056) = -.017, which was significant ($\hbox {z }= -2.73$, $\hbox {p }= .006$).

**Table 7:** Indirect and total effects for social distancing mediation model.

Type	Effect	Estimate	SE	95% CI	95% CI	$\beta$	z	p
				Lower	Upper			
Indirect	Financial Const. $\Rightarrow$ Neg PANAS $\Rightarrow$ Social Dist.	−0.00792	0.0029	−0.0136	−0.00224	−0.0165	−2.73	0.006
Component	Financial Const. $\Rightarrow$ Neg PANAS	0.68418	0.04264	0.6006	0.76774	0.296	16.05	<.001
	Neg PANAS $\Rightarrow$ Social Dist.	−0.01158	0.00418	−0.0198	−0.0034	−0.0559	−2.77	0.006
Direct	Financial Const. $\Rightarrow$ Social Dist.	−0.01497	0.00966	−0.0339	0.00396	−0.0313	−1.55	0.121
Total	Financial Const. $\Rightarrow$ Social Dist.	−0.0229	0.00924	−0.041	−0.00479	−0.0478	−2.48	0.013

Confidence intervals computed with method: Standard (Delta method).

Betas are completely standardized effect sizes.

In addition to negative affect, social distancing decisions in Spring 2020 were likely related to one’s beliefs about the spread of COVID-19 and one’s risk of contracting it. Thus, we hypothesized that beliefs about personal risk and national spread might also mediate the relationship between financial constraint and social distancing behavior. Because we previously assumed that negative affect mediated the relationship between financial constraint and personal risk / national spread, we developed a path model to test the multiple pathways from financial constraint to social distancing. Results showed that the indirect paths through national spread (p = .003) and negative affect (p = .018) were both significant. The indirect path through personal risk was not significant (p = .863). Full details of this analysis are in the supplementary materials.

## Discussion

Human rights groups^44^ and the United Nations^45^ have both highlighted the inequities of COVID-19: it is believed the poor are more susceptible to infection than the rich. This is consistent with previous research showing that individuals with lower SES are often at a greater risk of contracting infectious diseases such as influenza and pneumonia^1–3^. In addition, COVID-related job losses and pay cuts in Spring 2020 harmed the ability of many Americans to earn money and increased perceptions of financial constraint.

While the actual risk of contracting COVID-19 might be higher for those with lower SES, the psychological relationship between the economic crisis and beliefs and behaviors related to the COVID-19 health crisis have received only limited attention from psychologists. In this paper, we demonstrate that there was a meaningful psychological relationship between economic and health considerations at the start of the pandemic, illustrating the importance of this topic in understanding the crises. Specifically, we hypothesized that the psychological impacts of financial constraint, stemming in part from COVID-related job impacts, “spilled over” to influence beliefs about the co-occurring public health crisis. In line with the assumed relationship between SES and COVID-19 risks, we find that people reporting greater financial constraint and lower perceived SES (see replication study in the supplementary materials) believed they are more likely to be infected. Interestingly, this belief persisted after accounting for the risk of contracting COVID at work. Further, the relationship between financial constraint and beliefs about COVID-19 extended beyond personal risk to heightened perceptions of the national spread of the disease and increased social distancing behaviors. However, we note that the effect of financial constraint on these variables is small. For example, in the model predicting personal risk, the odds ratio for financial constraint is 1.09, meaning that a one point increase in financial constraint increases perceived personal risk by 1.09 times. Despite the effects being small, we believe our results demonstrate the existence of an important link between economic and health beliefs.

This raises the important question of why people’s financial vulnerability is correlated with their overall belief about the virus spread? In our models, financial constraint significantly predicted negative affect. In turn, negative affect predicted both perceptions of personal risk and national disease spread as well as social distancing behavior, accounting for much of the variance in the relationship between financial constraint and these types of risk perceptions and related behaviors.

Financial constraint, of course, is not the only factor that is likely to predict people’s beliefs about COVID-19 and social distancing behaviors. While there are many possible relevant factors (for example,^46^ examined the relationship between risk perceptions and beliefs about Covid-19 vaccines), we focus on two key factors that have received significant attention in the literature: political affiliation and local disease severity. The impact of partisanship on people’s health related behaviors (e.g., social distancing) and risk perceptions has received substantial attention in the media and academic work^12–14^. In line with those observations, we find self identified Democrats generally believed they were more likely to contract COVID-19 and that a higher proportion of the US would be infected than self identified Republicans. Further, self identified Democrats reported greater social distancing.

The other key factor we investigated was local disease severity. Interestingly, the association between objective disease prevalence and people’s risk perceptions was quite small. This runs counter to common epidemiological assumptions that people’s risk perceptions and behaviors are linked to disease prevalence. Chen^9^ assumed that people’s risk behavior depends directly on their perception of local disease prevalence. Epstein et al.^11^ modeled fear as an epidemic co-occurring with the medical epidemic where local disease conditions influence fear and fear influences behavior. Critically, while these (and other) studies assume local disease conditions influence risk perceptions (and thus behavior), we find that financial constraint and political affiliation have much stronger relationships. One possible reason that we do not see a relationship between local disease prevalence and beliefs is that the number of *reported* cases was very low at the time of data collection (about 0.2% of the US population). As this number increases, local disease prevalence might play a larger role in shaping people’s beliefs, particularly in cases when local disease severity is widely known and when there is high variation in this value. In fact, we do see a relationship between local cases and social distancing behavior, suggesting that people were sensitive to their local environments.

Importantly, the present research relies on the use of cross-sectional data, which limits our ability to draw strong causal inferences. Obtaining experimental data addressing issues of COVID-19 risk perception would be difficult, if not unethical, given the rapidly evolving nature of the public health and economic crises at the time. In the introduction, we outline a strong theoretical rationale in support of pathways from financial constraint to emotional distress and from emotional distress to risk perception. However, putting aside causality, our results indicate that financial constraint and emotional distress have substantial predictive utility independent of other commonly considered factors. Future research is needed to further confirm the direction of causality among these key variables.

In addition to being relevant to the COVID-19 public health and financial crises, our results may have broader implications for psychological science beyond COVID-19. A growing body of literature demonstrates that financial fragility and scarcity impact cognition and behavior^15–19,21,22^. Our research further illustrates this by showing financial constraint directly relates to a person’s emotional state, which is predictive of health beliefs and related behaviors. While some elements of these conclusions are undoubtedly linked to the COVID-19 crisis, others, particularly the relationships between financial constraint, emotions, and health perceptions, are likely more general. In sum, our research provides unique psychological insights into the experiences of people who feel financially vulnerable, both with respect to the COVID-19 health crisis and more broadly.

## Methods

### Participants

We recruited a nationally representative sample of 3,949 participants from Lucid^47^ in two separate cross-sectional waves of data, beginning on March 31, 2020 (n = 1987) and April 3, 2020 (n = 1962). Informed consent was obtained from all participants. We targeted 2,000 participants for each wave, prior to exclusions. Our aim was to have between 1000 and 1500 participants in each wave after exclusions. Sample size was predetermined before running the study, and we did not analyze data until all of the data had been collected. As described in the Results section, we had a total of 2,682 participants after exclusions. These participants ranged in age from 18-99 (*M*=50), 50% were male, with an average income of between $50,000-$59,999. The participant locations (by zip codes) are illustrated in the Supplementary Materials. This study was approved by the Institutional Review Board at the University of Chicago. All methods were carried out in accordance with relevant guidelines and regulations.

Participants were excluded based on the following criteria: (1) education level was not reported, (2) a valid zip code was not provided, (3) political affiliation was reported as ‘other’, and (4) invalid responses to three questions about predicting (remembering) the number of future (past) cases. For these questions, participants were provided clear instructions on appropriate responses (e.g., a number less than the US population of 329,000,000). Invalid responses on these questions likely indicate inattentive and disengaged participants. In total, there were 2,682 participants after the exclusions.

### Materials and procedure

After entering the survey and providing consent, participants answered a series of approximately 50 questions. These questions fell into six broad categories: psychological characteristics (e.g., emotional state, risk attitude), expectations about future disease spread (e.g., predictions about future spread, personal risk of infection), memories about past disease severity (e.g., recollections of past case numbers), financial fragility (e.g., financial constraint, COVID-related job risk), actions and decisions (e.g., social distancing), and demographics (e.g., political affiliation, age, zip code). The full list of questions can be found in the supplement.

We focus our analysis on several key questions. To examine beliefs about personal risk and national spread of COVID-19, we asked two questions: (1) How likely do you think it is that you will be infected with COVID-19 within the next year? and (2) What percent of the US population do you think will be infected with COVID-19 within the next year? The response scale had 21-points ranging from 0% to 100% and is provided in the supplement.

To examine financial constraint and COVID-19 related job impacts we asked participants the following questions: (1) “How financially constrained do you feel?”, on a 7-point scale from Not at all financially constrained, to Very financially constrained. (2) “To what extent does working at your current or most recent job put you at risk of contracting COVID-19?” on a 5-point scale from “My job puts me in extreme risk” to “My job does not put me in any risk”. (3) “To what extent has COVID-19 impacted your ability to earn money?” on a 5-point scale from “It has made it much harder for me to earn money” to “It has made it much easier for me to earn money”. For the analyses, questions 2 and 3 were reverse coded to match the coding of the financial constraint question (i.e., higher numbered responses indicate increased COVID-19 job impacts).

To examine recent emotional state, participants answered a short-form of the Positive and Negative Affect Schedule (PANAS)^48^: “Thinking about yourself and how you feel today, to what extent do you feel?” Participants responded using a 5-point scale from 1 = Not at all to 5 = Extremely for the following items: Upset, Hostile, Alert, Ashamed, Inspired, Nervous, Determined, Attentive, Afraid, and Active.

To measure social distancing, participants were asked “Thinking about everything you’ve done in the past 24 hours, which of the following comes closest to describing your in-person contact with people outside your household?” There were five response options: (1) Completely isolated yourself, having no contact with people outside your household, (2) Mostly isolated yourself, having very little contact with people outside your household, (3) Partially isolated yourself, having some contact with people outside your household, (4) Isolated yourself a little, still having a fair amount of contact with people outside your household, and (5) Did not make any attempt to isolate yourself from people outside your household.

Finally, participants completed demographic measures including zip-code, income, age, and education, and they selected their political affiliation from the following options: Republican, Lean Republican, Independent, Lean Democrat, Democrat, Other.

## Supplementary Information

Supplementary Information 1.

## Data Availability

The data reported in this manuscript were collected as part of a larger cross-sectional survey. De-identified data for the four waves used in this paper along with the data analysis scripts are posted at https://osf.io/rdtgk/. The materials used in these studies are available in the supplementary materials.
