# Supplementary material for: Financial constraint and perceptions of COVID-19
Source: Sci Rep. 2023 Mar 1;13:3432. doi: 10.1038/s41598-023-30118-9 (PMC9978024; doi:10.1038/s41598-023-30118-9)
Supplement: Supplementary file 1 — Supplementary Information 1. [file 41598_2023_30118_MOESM1_ESM.pdf]

Supplemental Data Analyses for “Financial Constraint and Perceptions of COVID-19”

Main Study

Method

Participants. Supplementary Figure S1 shows the locations of participants in the main study.

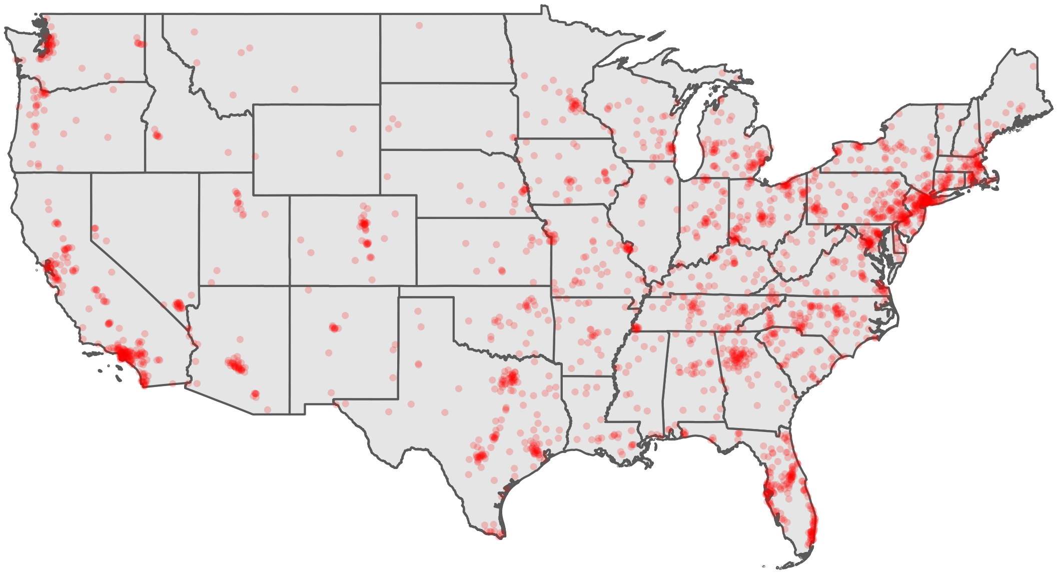

Figure S1. : Participant locations (by zipcode) from the main sample. The map was created using Plotly (version 5.9.0), an open source graphing library in Python, which can be accessed at https://plotly.com/python/maps/

Results

Predictors of social distancing. We hypothesized that, in addition to negative affect, beliefs about personal risk and nation spread mediate the relationship between financial constraint and social distancing behavior. Because we previously assumed that negative affect mediated the relationship between personal risk / national spread and financial constraint, we developed a path model to test the multiple pathways from financial constraint to social distancing. The results of

this analysis is shown in Supplementary Table S1. We see that the indirect paths through national spread and negative affect are both significant. The indirect path through personal risk is not.

Table S1:: Indirect and total effects for social distancing path model.

Type	Effect	Estimate	SE	95% CI Lower	95% CI Upper	β	z	p
Indirect	Fin Const ⇒ Neg ⇒ Soc Dist	-0.00694	0.00293	-0.01269	-0.0012	-0.01449	-2.368	0.018
	Fin Const ⇒ Nat Spread ⇒ Soc Dist	-0.00339	0.00114	-0.00563	-0.00116	-0.00708	-2.973	0.003
	Fin Const ⇒ Per Risk ⇒ Soc Dist	4.08E-05	2.36E-04	-4.22e4	5.03E-04	8.52E-05	0.173	0.863
	Fin Const ⇒ Neg ⇒ Nat Spread ⇒ Soc Dist	-0.00106	3.56E-04	-0.00176	-3.63e4	-0.00221	-2.98	0.003
	Fin Const ⇒ Neg ⇒ Per Risk ⇒ Soc Dist	7.81E-05	4.47E-04	-7.99e4	9.55E-04	1.63E-04	0.175	0.861
Component	Fin Const ⇒ Neg	0.68418	0.04264	0.60061	0.76774	0.29598	16.047	<.001
	Neg ⇒ Soc Dist	-0.01015	0.00424	-0.01845	-0.00184	-0.04897	-2.394	0.017
	Fin Const ⇒ Nat Spread	0.23242	0.04801	0.13833	0.32651	0.09661	4.842	<.001
	Nat Spread ⇒ Soc Dist	-0.01459	0.00387	-0.02219	-0.007	-0.0733	-3.767	<.001
	Fin Const ⇒ Per Risk	0.07976	0.06348	-0.04465	0.20418	0.02503	1.257	0.209
	Per Risk ⇒ Soc Dist	5.12E-04	0.00293	-0.00523	0.00625	0.00341	0.175	0.861
	Neg ⇒ Nat Spread	0.10623	0.02077	0.06552	0.14693	0.10207	5.115	<.001
	Neg ⇒ Per Risk	0.2231	0.02746	0.16928	0.27693	0.16181	8.124	<.001
Direct	Fin Const ⇒ Soc Dist	-0.01162	0.00968	-0.03059	0.00735	-0.02426	-1.201	0.23
Total	Fin Const ⇒ Soc Dist	-0.0229	0.00924	-0.041	-0.00479	-0.04781	-2.478	0.013

Note. Confidence intervals computed with method: Standard (Delta method)
Note. Betas are completely standardized effect sizes

Replication Study

Method

Participants. We collected a third cross-sectional wave of data, beginning on April 7, 2020 (n = 2025), and a fourth wave of data, beginning on April 10, 2020 (n = 2080), from Lucid. As in the main study, we targeted a nationally representative sample of 2,000 participants for this wave, prior to exclusions. Our aim was to have between 1000 and 1500 participants after exclusions. Sample size was predetermined before running the study, and we did not analyze data until all of the data had been collected. As described in the Results section, we had a total of 2,361 participants after exclusions. These participants ranged in age from 18-90 (M=44), 50% were male, with an average income of between $50,000-$59,999. Supplementary Figure S2 shows the locations of participants in the replication study. This study was approved by the Institutional Review Board at the University of Chicago.

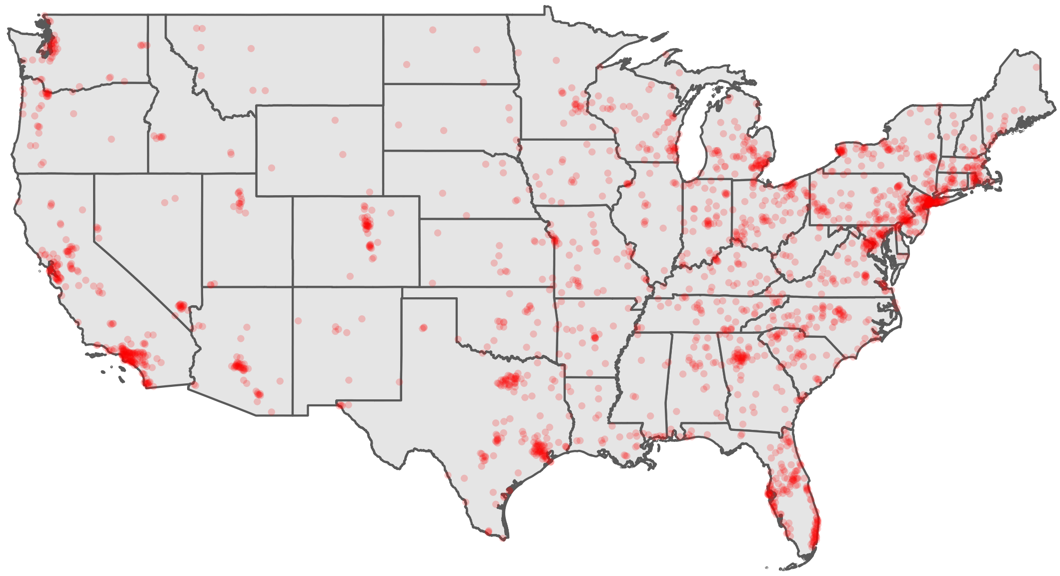

Figure S2. : Participant locations (by zipcode) from replication sample. The map was created using Plotly (version 5.9.0), an open source graphing library in Python, which can be accessed at https://plotly.com/python/maps/

Materials and Procedure. The materials and procedure were identical to the main study, except for the inclusion of one new question on perceived SES. For this question, participants were shown an image of a ladder and told “Think of this ladder as representing where people stand in their communities. People define community in different ways; please define it in whatever way is most meaningful to you. At the top of the ladder are the people who have a the highest standing in their community. At the bottom are the people who have the lowest standing in their community. Where would you place yourself on this ladder? Please choose a number on the rung where you think you stand at this time in your life, relative to other people in your community.” Responses were reported on a 10-point scale where a response of 1 was associated with the lowest rung on the ladder and a response of 10 was associated with the highest rung on the ladder.

Results

Participants were excluded based on the following criteria, matching the exclusion criteria from the main study: (1) education level was not reported, (2) a valid zip code was not provided, (3) political affiliation was reported as 'other', and (4) invalid responses to three questions about predicting (remembering) the number of future (past) cases. For these questions, participants were provided clear instructions on appropriate responses (e.g., a number less than the US population of 329,000,000). Invalid responses on these questions likely indicate inattentive and disengaged participants. In total, there were 2,361 participants after the exclusions.

Predictors of financial constraint. An ordinal logistic regression was calculated to predict financial constraint based on seven predictors: perceived SES, impact on earnings, COVID-19 job risks, income, age, education, and political affiliation. Results are shown in Supplementary Table S2

Table S2:: Results for ordinal regression model predicting feelings of financial constraint.

Predictor	Estimate	95% CI Lower	95% CI Upper	SE	Z	p	Odds ratio
Impact on Earnings	0.80789	0.7183	0.89827	0.0459	17.603	<.001	2.243
Job Risks	0.13007	0.0769	0.18332	0.02714	4.793	<.001	1.139
Income	-0.09251	-0.1148	-0.07025	0.01137	-8.135	<.001	0.912
Age	-0.00599	-0.0106	-0.00143	0.00233	-2.574	0.01	0.994
Education	0.01288	-0.0276	0.05334	0.02064	0.624	0.532	1.013
Perceived SES	-0.22305	-0.263	-0.18333	0.02032	-10.979	<.001	0.8
Political Affiliation:							
Lean Rep – Rep	0.18471	-0.093	0.46298	0.1418	1.303	0.193	1.203
Ind – Rep	0.17703	-0.0283	0.38249	0.10478	1.689	0.091	1.194
Lean Dem – Rep	0.3179	0.088	0.54828	0.1174	2.708	0.007	1.374
Dem – Rep	0.22368	0.0186	0.42904	0.10469	2.137	0.033	1.251

Predictors of beliefs about personal risk of contracting COVID-19. An ordinal logistic regression was calculated with eight predictors: perceived SES, financial constraint, political affiliation, local cases, COVID-19 job risks, income, age, and education. Results are shown in Supplementary Table S3.

We hypothesized that negative affect mediates the relationship between financial constraint and beliefs about personal risk of contracting COVID-19. Negative affect was calculated as the

Table S3:: Results for ordinal regression model predicting beliefs about personal risk.

Predictor	Estimate	95% CI Lower	95% CI Upper	SE	Z	p	Odds ratio
Financial Constraint	0.15894	0.11669	0.2013	0.02158	7.364	<.001	1.172
Job Risks	0.06741	0.01695	0.1179	0.02576	2.617	0.009	1.07
Income	0.08819	0.06603	0.1104	0.01133	7.786	<.001	1.092
Age	-0.00486	-0.00925	-4.83e4	0.00224	-2.175	0.03	0.995
Education	0.07536	0.03591	0.1149	0.02014	3.741	<.001	1.078
Local Cases (log)	0.04788	-0.1293	0.2251	0.09038	0.53	0.596	1.049
Perceived SES	-0.0687	-0.10715	-0.0303	0.0196	-3.506	<.001	0.934
Political Affiliation:							
Lean Rep – Rep	0.29846	0.03093	0.5666	0.1367	2.183	0.029	1.348
Ind – Rep	0.22649	0.0246	0.4286	0.10304	2.198	0.028	1.254
Lean Dem – Rep	0.38121	0.15389	0.6088	0.11603	3.285	0.001	1.464
Dem – Rep	0.32659	0.12531	0.5281	0.10273	3.179	0.001	1.386

sum of the five negative affect items on the short-form of the PANAS (i.e., upset, hostile, ashamed, nervous, and afraid). The indirect and total effects from the mediation analysis are show in Supplementary Table S4. As shown in the table, the standardized regression coefficient between financial constraint and negative affect was statistically significant (b = .285, z = 14.47, $p < .001$). The standardized regression coefficient between negative affect and personal risk (b = .111, z = 5.24, $p < .001$) was also significant. The standardized indirect effect was (.285)(.111) = .032, which was significant (z = 4.92, $p < .001$).

Table S4:: Indirect and total effects for personal risk mediation model.

Type	Effect	Estimate	SE	95% CI Lower	95% CI Upper	β	z	p
Indirect	Financial Const. ⇒ Neg PANAS ⇒ Per Risk	0.101	0.0205	0.0608	0.141	0.0316	4.92	<.001
Component	Financial Const. ⇒ Neg PANAS	0.629	0.0435	0.5435	0.714	0.2853	14.47	<.001
	Neg PANAS ⇒ Personal Risk	0.161	0.0307	0.1006	0.221	0.1108	5.24	<.001
Direct	Financial Const. ⇒ Personal Risk	0.336	0.0676	0.2032	0.468	0.105	4.97	<.001
Total	Financial Const. ⇒ Personal Risk	0.437	0.0652	0.309	0.565	0.1366	6.7	<.001

Note. Confidence intervals computed with method: Standard (Delta method)
Note. Betas are completely standardized effect sizes

Predictors of beliefs about the national spread of COVID-19. Next, we examined the factors that predict people's beliefs about the national spread of COVID-19. Similar to the analyses described above, we examine eight predictors: perceived SES, financial constraint, political affiliation,

local cases, COVID-19 job risks, income, age, and education. Results are shown in Supplementary Table S5.

Table S5:: Results for ordinal regression model predicting beliefs about national spread.

Predictor	Estimate	95% CI Lower	95% CI Upper	SE	Z	p	Odds ratio
Financial Constraint	0.12669	0.0851	0.16843	0.02127	5.957	<.001	1.135
Job Risks	-0.01498	-0.0645	0.03458	0.02529	-0.592	0.554	0.985
Income	0.02122	-6.62e4	0.04313	0.01117	1.9	0.057	1.021
Age	-0.0089	-0.0133	-0.00449	0.00225	-3.955	<.001	0.991
Education	-0.01872	-0.0581	0.02062	0.02007	-0.933	0.351	0.981
Local Cases (log)	0.01962	-0.1581	0.19735	0.09066	0.216	0.829	1.02
Perceived SES	-0.0293	-0.0675	0.00887	0.01948	-1.504	0.133	0.971
Political Affiliation:							
Lean Rep – Rep	0.16881	-0.0995	0.43674	0.13691	1.233	0.218	1.184
Ind – Rep	0.2192	0.0159	0.42269	0.10378	2.112	0.035	1.245
Lean Dem – Rep	0.24491	0.0171	0.47291	0.11628	2.106	0.035	1.278
Dem – Rep	0.41497	0.2157	0.61448	0.10171	4.08	<.001	1.514

Similar to before, we hypothesized that negative affect mediates the relationship between financial constraint and beliefs about the national spread of COVID-19. Negative affect was calculated as before. The indirect and total effects from the mediation analysis are show in Supplementary Table S6. As shown in the table, the standardized regression coefficient between financial constraint and negative affect was statistically significant (b = .285, z = 14.47, $p < .001$), as was the standardized regression coefficient between negative affect and national spread (b = .057, z = 2.69, p = .007). The standardized indirect effect was (.285)(.057) = .016, which was significant (z = 2.64, p = .008).

Table S6:: Indirect and total effects for national spread mediation model.

Type	Effect	Estimate	SE	95% CI Lower	95% CI Upper	β	z	p
Indirect	Financial Const. ⇒ Neg PANAS ⇒ Nat. Spd	0.0401	0.0152	0.0104	0.0699	0.0163	2.64	0.008
Component	Financial Const. ⇒ Neg PANAS	0.6287	0.0435	0.5435	0.7139	0.2853	14.47	<.001
	Neg PANAS ⇒ Nat. Spread	0.0638	0.0237	0.0173	0.1103	0.057	2.69	0.007
Direct	Financial Const. ⇒ Nat. Spread	0.3351	0.0523	0.2326	0.4376	0.1358	6.41	<.001
Total	Financial Const. ⇒ Nat. Spread	0.3752	0.0502	0.2768	0.4736	0.152	7.47	<.001

Note. Confidence intervals computed with method: Standard (Delta method)
Note. Betas are completely standardized effect sizes

Predictors of social distancing. In our final set of analyses, we examined the factors that predict people's social distancing behavior. Similar to before, we examined eight predictors: perceived SES, financial constraint, political affiliation, local cases, COVID-19 job risks, income, age, and education. Results are shown in Supplementary Table S7.

Table S7:: Results for ordinal regression model predicting social distancing.

Predictor	Estimate	95% CI Lower	95% CI Upper	SE	Z	p	Odds ratio
Financial Constraint	-0.08951	-0.13392	-0.0452	0.02263	-3.9555	<.001	0.914
Job Risks	0.17353	0.12018	0.22704	0.02726	6.3661	<.001	1.189
Income	-0.02601	-0.04949	-0.00258	0.01197	-2.174	0.03	0.974
Age	0.00261	-0.0021	0.00732	0.0024	1.0871	0.277	1.003
Education	0.01953	-0.02242	0.06152	0.02141	0.9124	0.362	1.02
Local Cases (log)	-0.17373	-0.36263	0.01482	0.09627	-1.8046	0.071	0.841
Perceived SES	-0.022	-0.0627	0.01869	0.02076	-1.0597	0.289	0.978
Political Affiliation:							
Lean Rep – Rep	0.06263	-0.21885	0.34357	0.14343	0.4367	0.662	1.065
Ind – Rep	-0.00396	-0.2182	0.21021	0.10927	-0.0363	0.971	0.996
Lean Dem – Rep	-0.00735	-0.24911	0.23409	0.12324	-0.0596	0.952	0.993
Dem – Rep	-0.32715	-0.54071	-0.11406	0.10882	-3.0063	0.003	0.721

Similar to beliefs about personal risk and the national spread of COVID-19, we hypothesized that negative affect mediates the relationship between financial constraint and social distancing behavior. Negative affect was calculated as before. The indirect and total effects from the mediation analysis are show in Supplementary Table S8. As shown in the table, the standardized regression coefficient between financial constraint and negative affect was statistically significant (b = .285, z = 14.47, $p < .001$), and the standardized regression coefficient between negative affect and social distancing was marginally significant (b = -0.039, z = -1.83, p = .068). The standardized indirect effect was (.285)(-0.039) = -.011, which was marginally significant (z = -1.81, p = .070).

In addition to negative affect, we hypothesized that beliefs about personal risk and national spread mediate the relationship between financial constraint and social distancing behavior. Because we previously assumed that negative affect mediated the relationship between personal risk / national spread and financial constraint, we developed a path model to test the multiple pathways from financial constraint to social distancing. The results of this analysis is shown in Supplementary Table S9. We see that the indirect path through national spread is significant and the indirect

Table S8:: Indirect and total effects for social distancing mediation model.

Type	Effect	Estimate	SE	95% CI Lower	95% CI Upper	β	z	p
Indirect	Fin Const. ⇒ Neg PANAS ⇒ Soc Dist.	-0.00572	0.00316	-0.0119	4.66E-04	-0.0112	-1.81	0.07
Component	Financial Const. ⇒ Neg PANAS	0.6287	0.04346	0.5435	0.71388	0.2853	14.47	<.001
	Neg PANAS ⇒ Social Dist.	-0.0091	0.00498	-0.0189	6.64E-04	-0.0391	-1.83	0.068
Direct	Financial Const. ⇒ Social Dist.	-0.0219	0.01097	-0.0434	-3.94e4	-0.0428	-2	0.046
Total	Financial Const. ⇒ Social Dist.	-0.02762	0.01053	-0.0482	-0.00699	-0.0539	-2.62	0.009

Note. Confidence intervals computed with method: Standard (Delta method)
Note. Betas are completely standardized effect sizes

path through negative affect is marginally significant. The indirect path through personal risk is not significant.

Table S9:: Indirect and total effects for social distancing path model.

Type	Effect	Estimate	SE	95% CI Lower	95% CI Upper	β	z	p
Indirect	Fin Const ⇒ Neg ⇒ Soc Dist	-0.0052	0.00317	-0.01141	0.001	-0.01016	-1.643	0.1
	Fin Const ⇒ Nat Spread ⇒ Soc Dist	-0.0046	0.00161	-0.00776	-0.00144	-0.00899	-2.855	0.004
	Fin Const ⇒ Per Risk ⇒ Soc Dist	1.23E-04	0.00112	-0.00207	0.00232	2.39E-04	0.109	0.913
	Fin Const ⇒ Neg ⇒ Nat Spread ⇒ Soc Dist	-5.51e4	2.71E-04	-0.00108	-2.03e5	-0.00108	-2.035	0.042
	Fin Const ⇒ Neg ⇒ Per Risk ⇒ Soc Dist	3.69E-05	3.37E-04	-6.23e4	6.97E-04	7.20E-05	0.109	0.913
Component	Fin Const ⇒ Neg	0.6287	0.04346	0.54352	0.71388	0.28535	14.467	<.001
	Neg ⇒ Soc Dist	-0.00828	0.00501	-0.01809	0.00153	-0.03562	-1.654	0.098
	Fin Const ⇒ Nat Spread	0.33506	0.05231	0.23255	0.43758	0.13575	6.406	<.001
	Nat Spread ⇒ Soc Dist	-0.01374	0.00431	-0.02218	-0.0053	-0.06622	-3.189	0.001
	Fin Const ⇒ Per Risk	0.33574	0.06761	0.20322	0.46825	0.10502	4.966	<.001
	Per Risk ⇒ Soc Dist	3.65E-04	0.00333	-0.00617	0.0069	0.00228	0.11	0.913
	Neg ⇒ Nat Spread	0.06382	0.02374	0.01729	0.11035	0.05697	2.688	0.007
	Neg ⇒ Per Risk	0.1607	0.03069	0.10055	0.22084	0.11075	5.237	<.001
Direct	Fin Const ⇒ Soc Dist	-0.01742	0.0111	-0.03918	0.00434	-0.03401	-1.569	0.117
Total	Fin Const ⇒ Soc Dist	-0.02762	0.01053	-0.04825	-0.00699	-0.05393	-2.624	0.009

Note. Confidence intervals computed with method: Standard (Delta method)
Note. Betas are completely standardized effect sizes

Survey Materials

There were 56 questions in the survey. They are listed below in the order they were presented to participants.

1. In this first set of 10 decisions, imagine that you are given a chance to earn a cash prize today. For each decision, you will choose between playing a Gamble and a sure thing.

Option A	Option B
90% Chance of $100	$50 for sure
80% Chance of $100	$50 for sure
70% Chance of $100	$50 for sure
60% Chance of $100	$50 for sure
50% Chance of $100	$50 for sure
40% Chance of $100	$50 for sure
30% Chance of $100	$50 for sure
20% Chance of $100	$50 for sure
10% Chance of $100	$50 for sure

2. Thinking about yourself and how you feel today, to what extent do you feel (1 = Not at all, 5 = Extremely)
   - Upset
   - Hostile
   - Alert
   - Ashamed
   - Inspired
   - Nervous
   - Determined
   - Attentive
   - Afraid
   - Active

3. Did you experience the following feelings during a lot of the day **yesterday**? (Yes/No)
   - Enjoyment
   - Physical Pain
   - Worry
   - Sadness
   - Stress
   - Anger

4. To what extent do you agree or disagree with the following statements? (Strongly agree / Somewhat agree / Neither agree nor disagree / Somewhat disagree / Strongly disagree)
   1. When I make plans, I am almost certain that I can make them work
   2. Getting people to do the right things depends upon ability; luck has nothing to do with It.

3. What happens to me is my own doing
4. Many of the unhappy things in people's lives are partly due to bad luck
5. Getting a good job depends mainly on being in the right place at the right time
6. Many times I feel that I have little influence over the things that happen to me
7. My financial situation depends on my control of the situation

5. Today, are you **more** concerned with the health consequences or economic consequences of COVID-19?
   - Definitely health consequences
   - concerned about health and economic
   - Definitely economic consequences

6. There are currently 329,000,000 people in the US. As of this morning [Date], [XXX] people **have tested positive** for COVID-19 in the **US**.

How many **total** people do you think will have tested positive for COVID-19 in **1 week from now** in the **US**?

Please enter a number between [XXX] and 329,000,000. Please write out your number completely and do not abbreviate it (e.g., ten thousand is 10,000; one hundred thousand is 100,000; and 1 million is 1,000,000).

7. How many **additional** people do you think will have tested positive for COVID-19 **over the next 1 week** in the **US**?

   Please enter a number in the drop box below. For example, if there were currently 100,000 cases and you expected there would be 105,000 in 1 week, the increase would be 5,000, and you would select "4,001-5,000" from the drop box below.

8. How many **total** people do you think will have tested positive for COVID-19 in **2 weeks from now** in the **US**?

   Please enter a number between [XXX] and 329,000,000. Please write out your number completely and do not abbreviate it (e.g., ten thousand is 10,000; one hundred thousand is 100,000; and 1 million is 1,000,000).

9. How many **additional** people do you think will have tested positive for COVID-19 **over the next 2 weeks** in the **US**?

   Please enter a number in the drop box below. For example, if there were currently 100,000 cases and you expected there would be 105,000 in 2 weeks, the increase would be 5,000, and you would select "4,001-5,000" from the drop box below.

10. How likely do you think it is that <u>**you**</u> will have been infected with COVID-19 <u>**within the next year**</u>? (0% means you think you will definitely not have been infected, 100% means you think you will definitely have been infected.

Reponses were on a 21-point scale:

✓ 0%
0.5%
1%
2%
3%
4%
5%
6%
7%
8%
9%
10%
20%
30%
40%
50%
60%
70%
80%
90%
100%

11. What percent **<u>of the US population</u>** do you think will have been infected with COVID-19 **<u>within the next year</u>**? (0% means you think no one in the US will have been infected, 100% means you think everyone in the US will have been infected) There are currently 329,000,000 people in the US. As of this morning [Date], [XXX] people have tested positive for COVID-19 in the US.

    Responses were on a 21-point scale same as above.

12. Thinking back to **one week ago**, approximately how many people in **total** do you think had tested positive for COVID-19 in the **US**? Please enter a number less than 329,000,000. Please write out your number completely and do not abbreviate it (e.g., ten thousand is 10,000; one hundred thousand is 100,000; and 1 million is 1,000,000).

13. **Compare today to one week ago:** approximately how many more people do you think have tested positive for COVID-19 today when compared to **one week ago**, in **the US**?

Please enter a number in the drop box below. For example, if there were currently 100,000 cases and you thought there were only 95,000 1 week ago, the difference would be 5,000, and you would select "4,001-5,000" from the drop box below.

14. How surprised have you been by the change in the number of people who have tested positive for COVID-19 in the US in the past seven days?
    - Extremely surprised
    - Very surprised
    - Moderately surprised
    - Slightly surprised
    - Not at all surprised

15. How would you describe the way most Americans are dealing with COVID-19?
    - Most are underestimating the risks
    - Most are behaving appropriately
    - Most are overreacting to the actual risks

16. Are you more concerned about getting COVID-19 yourself, or spreading COVID-19 to others?
    - Much more concerned about myself
    - Somewhat more concerned about myself
    - Equally concerned about myself and others
    - Somewhat more concerned about others
    - Much more concerned about others

17. As researchers conducting a study, it is important for us to know that you are reading these questions carefully. To show us that you are reading, please type "clean" in the space beside "other" below.
    - Hand Sanitizer
    - Hand Soap
    - Cleansing Wipes
    - Face Masks
    - Other _____

18. How worried are you about the health consequences of COVID-19 **affecting you personally** over the next year? (1 = Not at all worried, 7 = Extremely worried)

19. How worried are you about the health consequences of COVID-19 affecting **people in your community** over the next year? (1 = Not at all worried, 7 = Extremely worried)

20. How worried are you about the health consequences of COVID-19 affecting **people in the US** over the next year? (1 = Not at all worried, 7 = Extremely worried)
21. How worried are you about the current **global** economy negatively affecting **your financial situation** over the next year? (1 = Not at all worried, 7 = Extremely worried)

22. How worried are you about the current **local** economy negatively affecting **your financial situation** over the next year? (1 = Not at all worried, 7 = Extremely worried)

23. How worried are you about the current **global** economy negatively affecting **your community** over the next year? (1 = Not at all worried, 7 = Extremely worried)

24. How worried are you about the current **local** economy negatively affecting **your community** over the next year? (1 = Not at all worried, 7 = Extremely worried)

25. Please consider the following items. First, think about how much you were willing to pay for this item at the beginning of January 2020 (prior to the COVID-19 outbreak in the US). Relative to that, approximately how much more or less would you be willing to pay now? Keep in mind that -100% means you would pay $0 now, while +100% means you would pay 2x as much now.
    - Non-perishable foods (e.g., pasta, dried beans, frozen vegetables)
    - Fresh fruits and vegetables (e.g., bananas, oranges, broccoli)
    - Cleaning supplies (e.g., soap, hand sanitizer, disinfecting wipes)
    - Clothing (e.g., pants, tops, shoes)
    - Technology (e.g., computers, phones, tablets)

26. Imagine that the government sent you a check for $1,000 that you cashed today. If you were to spend this money over the next month, how would you divide it across the following categories?

    - Housing (rent, mortgage) and utilities
    - Food
    - Medical and healthcare
    - Savings and investing
    - Debt repayments
    - Personal spending, recreation, and entertainment
    - Other (please specify)

27. To what extent do you worry about not having enough money to buy groceries next week? (1 = Not at all, 7 = Extremely much)

28. How difficult do you think it will be for you to pay your bills this month? (1 = Not difficult at all, 7 = Extremely difficult)

29. Thinking about everything you've done in the past 24 hours, which of the following comes closest to describing your in-person contact with people outside your household?

    - Completely isolated yourself, having no contact with people outside your household
    - Mostly isolated yourself, having very little contact with people outside your household
    - Partially isolated yourself, having some contact with people outside your household

- Isolated yourself a little, still having a fair amount of contact with people outside your household
- Did not make any attempt to isolate yourself from people outside your household

30. Within the past week, have you taken any of the following actions to help others? (check all that apply)

    - Volunteering time through a local organization (e.g., charity, church)
    - Helping friends or family directly with your time (e.g., running errands)
    - Lending money to friends or family that they will repay
    - Giving money to friends or family that they will keep
    - Donating money to local organizations (e.g., charity, church)
    - Donating money to national organizations (e.g., the Red Cross)
    - Helping local stores (e.g., purchasing gift-cards, donation campaigns)
    - Other (please specify)
    - I am not currently taking actions because I am unable
    - I am not currently taking actions because I do not want to
    - I am not currently taking actions, but plan to in the future

31. Within the past week, have you received help in any of the following forms? (check all that apply)
    - Receiving help form a local organization (e.g., charity, church)
    - Receiving help from a national organization (e.g., the Red Cross)
    - Receiving direct help from friends or family with regards to their time (e.g., running errands)
    - Borrowing money from friends or family with the plan to give it back
    - Receiving money from friends or family to keep
    - Receiving government aid (e.g., food stamps, unemployment payments, social security payments)
    - Other (please specify)
    - I am not receiving help

32. Discounts that you use
    We are interested in the types of discounts that you use. However, more influential than your discount preferences is whether you are taking the time to read these instructions.To indicate that you have read these instructions, please select the "other" option below and enter the word "food" in the available text box.
    - Rebates
    - Buy 2 get 1 free
    - Coupons
    - % off sales
    - Free shipping
    - Other

33. How are you coming up with money for daily activities? (check all that apply)
    - My current job

- I'm dependent on someone else (e.g., a spouse or parent)
- Drawing money from short-term savings
- Drawing money from long-term savings accounts (e.g., retirement accounts)
- Government assistance
- Borrowing from friends & family
- Borrowing on credit cards
- Borrowing through other source (please specify)
- Not able to keep up
- Other (please specify)
-

34. To what extent has COVID-19 impacted your ability to earn money?
    - It has made it much harder for me to earn money
    - It has made it somewhat harder for me to earn money
    - It has not impacted my ability to earn money
    - It has made it somewhat easier for me to earn money
    - It has made it much easier for me to earn money

35. To what extent does working at your current or most recent job put you at risk of contracting COVID-19?
    - My job puts me in extreme risk
    - My job puts me in a lot of risk
    - My job puts me in a moderate amount of risk
    - My job puts me in a little risk
    - My job does not put me in any risk

36. Have you lost your job as a result of the current economic downturn related to COVID–19? (Yes/No)

37. If you are currently employed, how confident are you that you will keep your job over the **next month**? (1 = Not at all Confident, 7 = Extremely Confident, Not Currently Employed)

38. If you are currently employed, how confident are you that you will keep your job over the **next 3 months**? (1 = Not at all Confident, 7 = Extremely Confident, Not Currently Employed)

39. What is your current employment status?
    - Employed
    - Self-employed
    - Unemployed looking for work
    - Unemployed not looking for work
    - Retired
    - Other

40. What job sector do you currently work in?
    - Healthcare

- Social assistance
- Hospitality and restaurants
- Government
- Construction
- Manufacturing
- Transportation
- Education
- Technology
- Other Services
- Other (please specify)

41. Please select your marital status
    - Married
    - Widowed
    - Divorced
    - Separated
    - Never Married
    - Other (please specify)

42. How financially constrained do you feel? (1 = Not at all financially, 7 = Very financially constrained)

43. Relative to others **in the US**, how would you view your current *financial* position?
    - Much worse than others
    - Somewhat worse than others
    - About the same as others
    - Somewhat better than other
    - Much better than others

44. Relative to others **in your community**, how would you view your current *financial* position?
    - Much worse than others
    - Somewhat worse than others
    - About the same as others
    - Somewhat better than other
    - Much better than others

45. Relative to **yourself one year ago**, how would you view your current *financial* position?
    - Much worse than others
    - Somewhat worse than others
    - About the same as others
    - Somewhat better than other
    - Much better than others

46. Relative to others **in the US**, how would you view your current *non-financial* position? (e.g., living situation, ability to have social interactions)

- Much worse than others
- Somewhat worse than others
- About the same as others
- Somewhat better than other
- Much better than others

47. Relative to others **in your community**, how would you view your current *non-financial* position? (e.g., living situation, ability to have social interactions)
    - Much worse than others
    - Somewhat worse than others
    - About the same as others
    - Somewhat better than other
    - Much better than others

48. Relative to **yourself one year ago**, how would you view your current *non-financial* position? (e.g., living situation, ability to have social interactions)
    - Much worse than others
    - Somewhat worse than others
    - About the same as others
    - Somewhat better than other
    - Much better than others

49. How many dependents do you currently have?

50. Please enter your age:

51. Please enter your gender: (Male/Female/Other)

52. What is your total pretax household income (in the past year)?
    - Less than $10,000
    - $10,000-$19,999
    - $20,000-$29,999
    - $30,000-$39,999
    - $40,000-$49,999
    - $50,000-$59,999
    - $60,000-$69,999
    - $70,000-$79,999
    - $80,000-$89,999
    - $90,000-$99,999
    - $100,000-$124,999
    - $125,000-$149,999
    - $150,000-$199,999
    - $200,000 or greater

53. What is your political affiliation?
    - Republican

- Lean Republican
- Independent
- Lean Democrat
- Democrat
- Other

54. Please enter your zip code:

55. Please let us know if you have any other thoughts about COVID-19 that you would like to share.

56. Please let us know if you have any comments about the survey or had any problems with the survey. Did you notice any errors or typos? Anything unclear?
